# Coffee Staining and Simulated Brushing Induced Color Changes and Surface Roughness of 3D-Printed Orthodontic Retainer Material

**DOI:** 10.3390/polym15092164

**Published:** 2023-05-01

**Authors:** Durgesh Bangalore, Abdullah M. Alshehri, Omar Alsadon, Samer M. Alaqeel, Omar Alageel, Majed M. Alsarani, Haitham Almansour, Obaid AlShahrani

**Affiliations:** Dental Health Department, College of Applied Medical Sciences, King Saud University, Riyadh 7268, Saudi Arabia; abmalshehri@ksu.edu.sa (A.M.A.); oalsadon@ksu.edu.sa (O.A.); salaqeel@ksu.edu.sa (S.M.A.); oalageel@ksu.edu.sa (O.A.); malsarani@ksu.edu.sa (M.M.A.); halmansour@ksu.edu.sa (H.A.);

**Keywords:** 3D printing, color, orthodontic retainer, stereolithography, surface roughness, tooth brushing

## Abstract

This in vitro study evaluated the influence of combined coffee staining and simulated brushing-induced color changes and surface roughness on 3D-printed orthodontic retainers. Specimens measuring 10 × 10 × 0.75 mm^3^ were obtained either by conventional vacuum forming or 3D printing at four print angulations (0°, 15°, 30°, and 45°) (n = 10). The prepared specimens were immersed in a coffee beverage and then mechanically brushed using a simulating device. The specimen’s color difference (ΔE) and surface roughness (Ra) were quantified using a spectrophotometer and a non-contact profilometer, respectively. The highest and lowest mean ΔE values were recorded for the 3D-printed-45° (4.68 ± 2.07) and conventional (2.18 ± 0.87) groups, respectively. The overall mean comparison of ΔE between the conventional and 3D-printed groups was statistically significant (*p* < 0.01). After simulated brushing, all groups showed a statistically significant increase in the Ra values (*p* < 0.01). The highest Ra was in the 3D-printed-45° (1.009 ± 0.13 µm) and conventional (0.743 ± 0.12 µm) groups, respectively. The overall ΔE of 3D-printed orthodontic retainers was not comparable to conventional VFRs. Among the different angulations used to print the retainers, 15° angulations were the most efficient in terms of color changes and surface roughness and were comparable to conventional VFRs.

## 1. Introduction

The implications of retention after properly performed orthodontic treatment are well understood. The periodontal and gingival tissues, masticatory muscles, occlusal stresses, and tongue, as well as any residual growth of the facial bones, can impact the stable position of the teeth [1,2]. The clinical purpose of retention, the final stage of active orthodontic therapy, is to maintain the teeth that have undergone orthodontic correction in a healthy, functional, and aesthetically pleasing position [1,3,4].

Insufficient data exist to determine which retention method is more appropriate for sustaining the outcome of orthodontic treatment [5]. Although many orthodontists prefer bonded retainers, a combination of fixed and removable retainers is most frequently used in orthodontic retention [6,7,8]. Since fixed retainers have a higher failure rate in the upper jaw than in the lower, most clinicians use maxillary removable and mandibular fixed retainers as their usual retention regimen for optimum relapse prevention [9]. The most popular removable orthodontic retainers, in general, are Hawley and vacuum-formed retainers (VFRs) [10,11]. The clinical performance, cost-effectiveness, aesthetic qualities, ease of fabrication, minimum modification needs, and patient acceptability of VFRs, however, have made them fairly popular [10,11]. The VFRs are made from a thermoplastic sheet that is heated and compressed inside a vacuum apparatus against the patient’s mold, then trimmed into a horseshoe shape, according to the manufacturer’s instructions [5]. In a recent clinical trial comparing bonded retainers versus VFRs after one year of use, bonded retainers accumulated more plaque and calculus than VFRs. They were also found to cause minimally worse gingival inflammation. However, there was no evidence of clinically significant, detrimental periodontal health issues [12].

Clinical orthodontics has been more effective due to the recent and rapid advancements in digital technology, especially computer-aided design (CAD) and computer-aided manufacturing (CAM) tools [13,14]. There are two main processing methods for CAD–CAM systems: subtractive manufacturing (milling) and additive manufacturing (3D printing). Three-dimensional printing fabricates 3D structures by connecting material from a 3D model layer by layer [15,16]. This advanced technique makes it feasible to create dental and orthodontic appliances directly from 3D models, which lessens the workload for dental technologists and clinicians [3,17]. Stereolithography (SLA) is the first and most well-known method of 3D printing, which uses a bath of methacrylate-based photocurable resin, a platform for creating models, and an ultraviolet laser to polymerize the resin. Although the SLA’s core technology has remained unchanged, recent advancements have produced a new generation of more compact, affordable, and productive printers than the initial SLA model [15,16].

In recent years, 3D printing technology has been used to fabricate orthodontic retainers from photocurable resin [14,15,18,19,20,21,22,23]. In 2014, Nasef et al. [23] reported the direct printing of an opaque retainer from a cone beam computed tomography scanned model and found no clinically significant differences between the 3D-printed and the VFRs [22]. In contrast to conventional VFRs and commercially available VFRs, 3D-printed retainers showed the most significant variation from the original reference models, according to a recent study by Cole et al. [15], yet the deviation was still within the clinically acceptable 0.5 mm range. Williams et al. [14] reported that 3D-printed retainers were precise within 0.25 mm at incisal edges and the cusp tips compared to the digital reference file. The smooth facial surfaces, however, did not meet clinical acceptability. The authors also demonstrated that print angulations impacted the resin cost and usage.

There is still some controversy regarding the ideal time frame for using orthodontic retainers. However, it has been demonstrated that periodontal apparatus regeneration may require up to 12 months to support the tooth in its new position appropriately. Relapse is almost certain to occur eventually without orthodontic retention [5]. As a result, most clinicians frequently recommend permanent retention [5,6]. Typically, patients are instructed to wear their aligners all the time, except for eating, drinking anything other than water, and washing or flossing their teeth [5]. Yet, many patients do not show complete compliance [6,24] and consume colorants with their appliances regardless of their orthodontists or the manufacturer’s instructions [6]. Long-term retainer use has been associated with a propensity for bacterial growth on the fitting surface. Retainers can harbor opportunistic pathogens such as Candida albicans and methicillin-resistant Staphylococcus aureus, which theoretically could cause a local or systemic infection [25,26].

Although the recommended cleaning procedures for orthodontic retainers are not yet established, they are crucial for guaranteeing their safe use [27]. The American Association of Orthodontists advises using a chemical cleanser or brushing the retainer twice daily with dentifrice [28]. On the contrary, the British Orthodontic Society recommends cleaning the retainers daily using cold water and advises against using toothpaste since it can roughen and alter their color [29]. Mechanical, chemical, or a combination of the two cleaning methods can be used to maintain the hygiene of the retainer [26,27,30,31]. The most popular method of cleaning dentures is with a toothbrush and dentifrice or tap water since it is easy to use, inexpensive, and effective at eliminating organic deposits [27,31,32,33,34].

Nevertheless, brushing with dentifrices has been reported to significantly impact the wear and roughness of restorative and prosthetic materials. Dental appliances are subjected to wear and roughness, which promote biofilm formation, discoloration, loss of surface details, and diminished gloss [16,17,18]. The final effect on material roughness relies on the applied load and brushing duration [34]. The color stability of clear retainers remains a significant consideration for both patients and clinicians [30]. These clear retainers are popular among adults who want a more aesthetic option with less metal exposure [27]. Patient adherence to retainer wear may be hampered by material degradation, surface roughness, and discoloration [27].

The precision and properties of 3D-printed resin materials are influenced by many factors, including resin type, printer used, surface treatment, and printing angulations [35]. Although the printing angulation of 3D-printed resin materials has found considerable importance among dental researchers, it is still unclear about the specific printing angulation to be applied for 3D printing [14,16,36,37,38,39,40,41,42]. Three-dimensional-printed orthodontic retainers are still a relatively new topic in the orthodontic specialty, necessitating a thorough analysis of the properties before considering it a viable option to conventional copolyester VFRs. To date, the effect of print angulation on the ΔE and Ra of 3D-printed orthodontic retainer materials remains unexplored. Therefore, this in vitro study aimed to evaluate the influence of printing angulation, coffee staining, and simulated brushing-induced color changes and surface roughness of 3D-printed orthodontic retainer and compare the outcome with the conventional thermoplastic orthodontic retainer. The first null hypothesis stated was that there is no significant difference in color and surface roughness between the 3D-printed and conventional orthodontic retainers. The second null hypothesis stated was that the four different printing angulations (0°, 15°, 30°, and 45°) would present no significant differences in the color and surface roughness when printing 3D-printed orthodontic retainers.

## 2. Materials and Methods

### 2.1. Specimen Preparation

**Figure 1:**
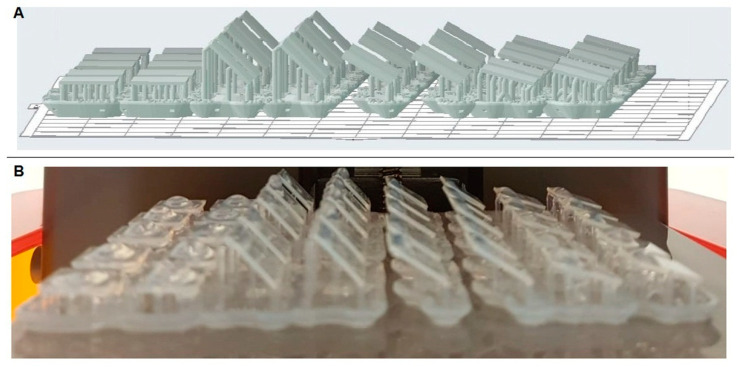
(**A**) STL file used for printing 3D specimens; (**B**) 3D-printed specimens at different angulations.

A sample size of 10 specimens per group was in accordance with a previous study [17]. The digital specimen measuring 10 × 10 × 0.75 mm^3^ was designed using CAD software, and the resulting STL file (Figure 1A) was imported into the PreForm print preparation software (Formlabs, Inc., Somerville, MA, USA). A total of 40 specimens were printed using a stereolithography (SLA) 3D printer (Formlabs, Inc., Somerville, MA, USA) and photopolymer resin (Dental LT Clear, Formlabs, Inc., Somerville, MA, USA). The chemical composition of the clear 3D print resin used in this study is 7,7,9 (or 7,9,9)-trimethyl-4,13-dioxo-3,14-dioxa-5,12- diazahexadecane-1,16-diyl bismethacrylate (50–75% *w*/*w*), 2-hydroxyethyl methacrylate (10–20% *w*/*w*), reaction mass of Bis(1,2,2,6,6-pentamethyl-4-piperidyl) sebacate and Methyl 1,2,2,6,6-pentamethyl-4-piperidyl sebacate (<10% *w*/*w*), diphenyl (2,4,6- trimethylbenzoyl)phosphine oxide (1–5% *w*/*w*), acrylic acid monoester with propane-1,2-diol (0.1–1% *w*/*w*), ethylene dimethacrylate (<10% *w*/*w*), 2-hydroxyethyl acrylate (0.1–1% *w*/*w*) and mequinol, 4-methoxyphenol, hydroquinone monomethyl ether (<0.1% *w*/*w*).

The specimens were printed at four print angulations: 0°, 15°, 30°, and 45° (n = 10) (Figure 1B) using a 100 mm layer-by-layer print thickness. Subsequently, the printed specimens were rinsed in two consecutive baths of 98% isopropyl alcohol for 5 min and rigorously dried with compressed air. Then the specimens were post-print cured for 30 min in a curing oven to ensure that the remaining monomers were cured. Print supports from printed specimens were removed carefully before color and roughness measurements.

The conventional retainer specimens were prepared by heating and vacuum forming of the copolyester thermoplastic sheets (Proform, Keystone Industries, Myerstown, PA, USA) over a steel block of a predetermined dimension using a thermoforming machine (Henry Schein Inc., Melville, NY, USA).

### 2.2. Coffee Staining and Simulated Brushing

Three teaspoons of coffee powder (Nescafe Classic, Nestle Middle East Manufacturing L.L.C.-Dubai, United Arab Emirates) were added to 250 mL of boiling water and continuously swirled for 10 min to prepare the coffee beverage. The resulting solution was filtered to remove the residue, then cooled to room temperature. The specimens were thoroughly cleaned under running water to remove debris before coffee staining. The specimens were continually immersed in Petri dishes containing ample amounts of the coffee beverage. The beverage was changed every 4 h over the 50 h total immersion period, which equated to two years of oral exposure [17,43].

Before simulating mechanical brushing, the specimens were rinsed with tap water and stored in distilled water at room temperature for 24 h. The simulating device (SD Mechatronik GMBH, Feldkirchen Westerham, Germany) had 12 separate slots, each holding a soft toothbrush (Colgate 360, Colgate-Palmolive, Istanbul, Turkey). A drop of adhesive monomer was used to glue the specimens to the dentifrice containers of the toothbrush simulator. A 1:1 mixture of dentifrice (Colgate Regular, Colgate-Palmolive Arabia L.T.D, Dammam, Kingdom of Saudi Arabia) and deionized water was prepared and poured into each of the 12 containers to immerse the specimens. The brushing parameters—356 rpm, 2N vertical load, 38 mm stroke length, and 35,600 brushing cycles—were applied. The brushing cycles were accomplished in 100 min, representing two years of oral exposure [17,44]. The brushing force was delivered in accordance with ISO 14569-1 specification, which specifies 0.5–2.5 N brushing forces [45]. According to the ADA recommendations, the toothbrushes were replaced after 4500 cycles, and the slurry was replenished during the brushing cycle [17].

### 2.3. Color

A bench-top UV light visible spectrophotometer (Konica Minolta Sensing Inc., Osaka, Japan) functioning at a wavelength between 360 and 740 nm was used to record the color of the specimens before and after coffee staining and brushing in the 3D Commission Internationale de l’Eclairege (CIE) Lab color space. Following the manufacturer’s recommendation, the spectrophotometer was calibrated against a white background. CIE color coordinates were measured using three-pulsed xenon lamps analogous to average daylight.

According to the International Organization for Standardization (ISO) technical specification ISO/TR 28642:2016 [46], the color difference (ΔE) of the specimen was quantified using the CIELab equation:

(1) $$\Delta E={\left[{\left(\Delta L^{*}\right)}^{2}+{\left(\Delta a^{*}\right)}^{2}+{\left(\Delta b^{*}\right)}^{2}\right]}^{1/2}$$

ΔL*, Δa*, and Δb* are the mean differences between the pre and post-coffee staining and brushing L*, a*, and b* values. L refers to the difference between light and dark, a is the difference in the red and green chromatic scale, and b is the difference in the quantity of yellow and blue.

In dentistry, the stated values for the CIELab 50% perceptibility threshold (PT) and acceptability threshold (AT) are ∆E = 1.2 and ∆E = 2.7, respectively. The ∆E values over the AT limit are clinically unacceptable [47].

In relating the ΔE to the clinical situation, the obtained values were converted to the National Bureau of Standards (NBS) units using Equation (2). The inferences of color changes as per NBS units are presented in Table 1 [48]:

NBS = ΔE × 0:92 (2)

### 2.4. Surface Roughness

The surface roughness of the specimens was measured using a non-contact optical profilometer (Bruker Contour GT, Tucson, AZ, USA). The profilometer includes a fully automated turret and a nano-lens atomic force microscopy (AFM) module. The specimen was mounted on the turret and scanned using white light interferometry without contacting the surface to measure the roughness. The profilometer equipped with Vision 64 software manages turret movement [35,49]. The specimen was scanned at three different regions, 2 mm apart, and the mean of the readings from the three scans corresponds to the roughness values of that particular specimen. The roughness average (Ra) in µm is used to quantify the roughness. Surface roughness measurement was performed at two intervals, before (Ra1) and after tooth brushing (Ra2).

### 2.5. Scanning Electron Microscopy (SEM) Analysis

A representative specimen from each group was observed under a scanning electron microscope (JEOL JSM-5900 LV SEM, Tokyo, Japan). Before the SEM analysis, the specimen was gold sputter coated for one minute in a coating machine (Q150R, Quorum Technologies, East Sussex, UK). The SEM micrographs were obtained at a magnification of ×1000, 10 kV in a vacuum, and a working distance of 10 µm.

**Figure 2:**
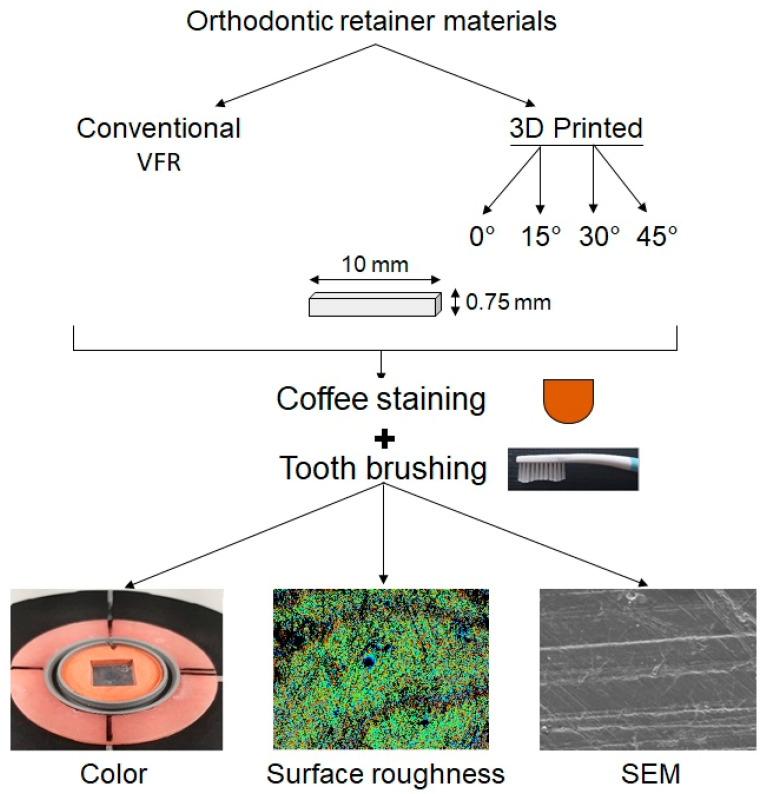
Flow chart illustrating the study process.

Figure 2 presents the flowchart illustrating the study process.

### 2.6. Statistical Analysis

Data were analyzed using SPSS analysis software (SPSS IBM v.22; IBM Corp, Armonk, NY, USA). Data followed a normal distribution (Shapiro–Wilk test; (*p* > 0.05)). Analysis of Variance (ANOVA) was used to find the significant difference between the groups. Post hoc Bonferroni multiple comparisons test was applied to find significant differences between the groups. Paired *t*-tests were used to compare the pre and post-brushing surface roughness for each group (α = 0.05).

## 3. Results

### 3.1. Color Change (ΔE)

**Figure 3:**
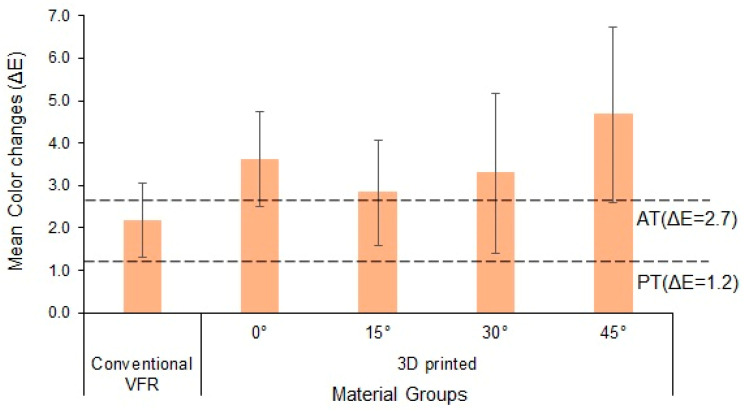
Mean color changes of the study groups. Bars indicate SD. The dotted lines indicate the perceptibility threshold (PT) and acceptability threshold (AT) limit.

Figure 3 presents the mean ΔE of the group according to the PT and AT limit. The higher and lower mean color changes were recorded in the 3D-printed-45° group (4.68 ± 2.07) and conventional VFRs group (2.18 ± 0.87), respectively. Among the tested materials, the ΔE values were above the AT limit except for the conventional VFRs, which exhibited ΔE values between the PT and AT limits.

Pairwise comparison for significant differences in ΔE among the study groups was carried out using Bonferroni multiple comparisons test (Table 2). A statistically significant difference was observed between conventional VFRs and 3D-printed-45° groups (*p* < 0.01). No significant difference was observed among the other groups (*p* > 0.05).

The overall mean comparison of ΔE between the conventional and 3D-printed groups showed a significant difference (*p* < 0.01) (Table 3). 

**Figure 4:**
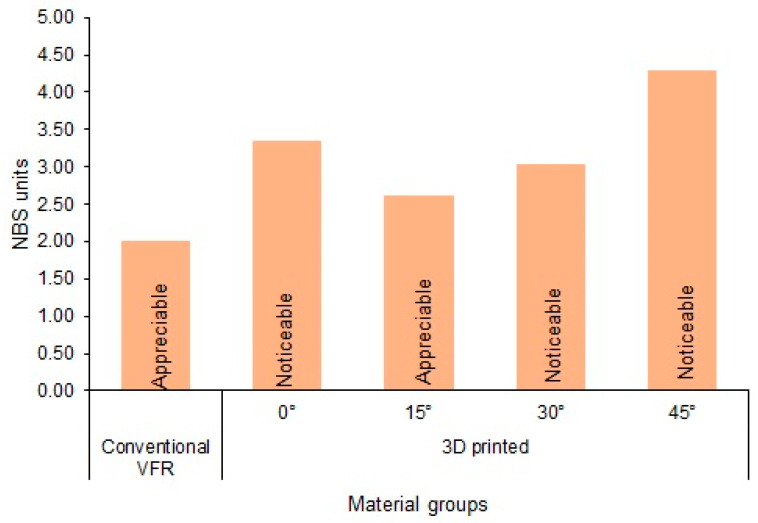
NBS inference of the ΔE values.

Figure 4 presents the NBS inference of the ΔE values. Conventional VFRs and 3D-printed-15° groups showed appreciable/marked color changes, while the remaining study groups showed noticeable/perceivable color changes.

### 3.2. Surface Roughness (Ra)

**Figure 5:**
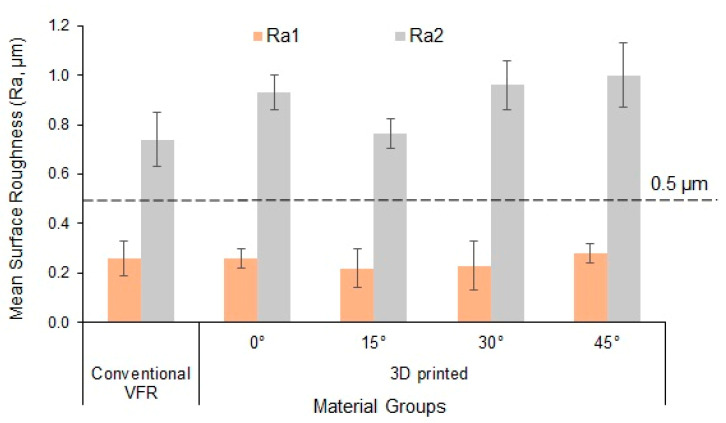
Mean surface roughness of the study groups. Bars indicate SD. The dotted line indicates the roughness threshold limit.

Figure 5 presents the mean Ra1 and Ra2 of the groups according to the roughness threshold limit. Mean Ra1 was found to be high in 3D-printed-45° (0.288 ± 0.05) and low in 3D-printed-15° (0.229 ± 0.08) groups. The difference in mean Ra1 amongst the groups was not statistically significant (*p* > 0.05).

After simulated brushing, all the groups showed increased Ra values. The highest and lowest Ra2 was in 3D-printed-45° (1.009 ± 0.13) and conventional VFRs (0.743 ± 0.12) groups, respectively.

Since the Ra1 between groups was statistically non-significant (*p* = 0.37), the pairwise comparison was not performed. Pairwise comparison for a significant difference in Ra2 among the groups was carried out using Bonferroni multiple comparisons test (Table 4). A statistically significant difference was observed between conventional VFRs and the 3D-printed—0° group (*p* < 0.01), conventional VFRs and the 3D-printed—30° group (*p* < 0.001), conventional VFRs and the 3D-printed—45° group (*p* < 0.001), 3D-printed—0°, and 3D-printed—15° group (*p* < 0.01), 3D-printed—15° and 3D-printed—30° group (*p* < 0.01) as well as between 3D-printed—15°, and 3D-printed—45° group (*p* < 0.001). No significant difference was observed among the other groups (*p* > 0.05).

Table 5 presents the mean comparison in roughness from Ra1 to Ra2. There was an increase in surface roughness from Ra1 to Ra2 in all the groups, and this increased roughness was statistically significant (*p* < 0.001).

The overall mean comparison of Ra2 between the conventional VFRs and 3D-printed groups was statistically significant (*p* < 0.01) (Table 6).

**Figure 6:**
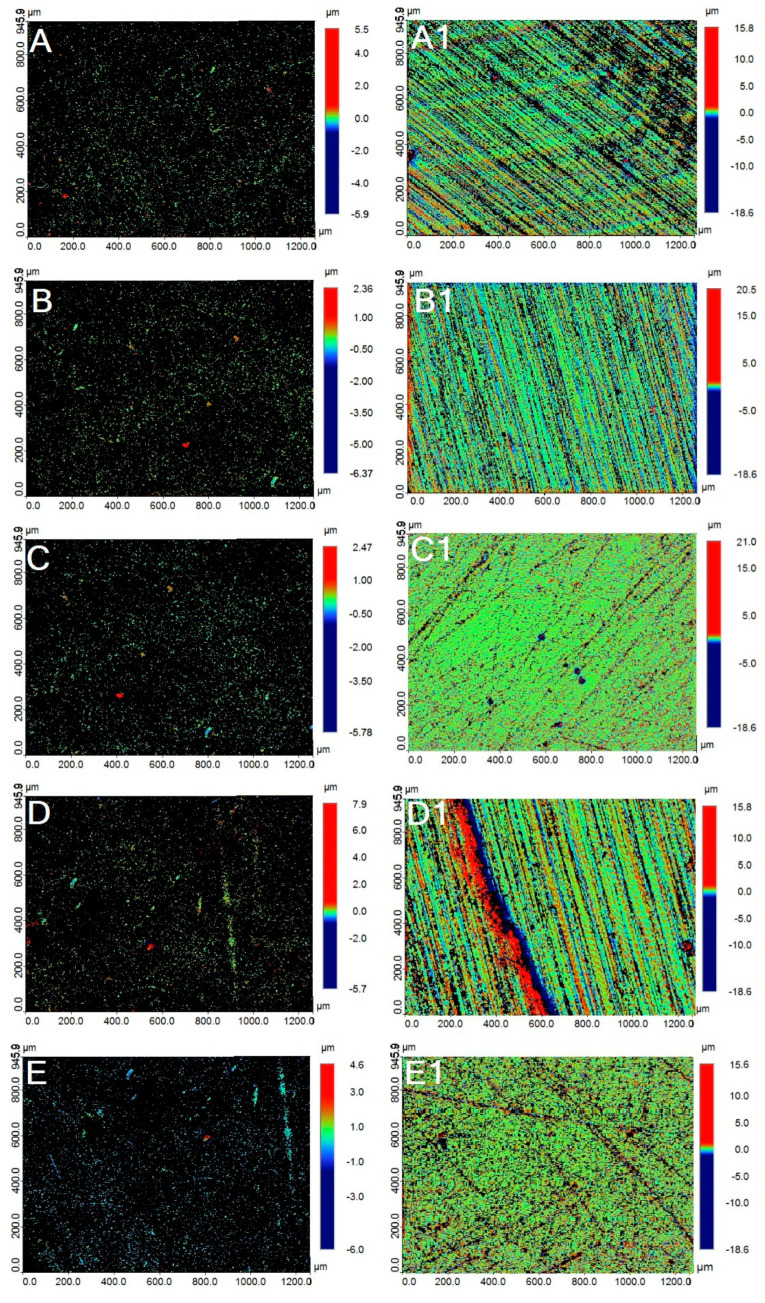
Representative profilometer images of the study groups obtained at Ra 1 (**A**–**E**) and Ra2 (**A1**–**E1**). (**A**,**A1**)-Conventional VFRs; (**B**,**B1**)-3D-printed—0°; (**C**,**C1**)-3D-printed—15°; (**D**,**D1**)-3D-printed—30° and; (**E**,**E1**)-3D-printed—45°.

Profilometer images of the representative specimen from each study group are presented in Figure 6. At Ra1, the specimens from the study groups (Figure 6A–E) showed an identical roughness profile, which is consistent with the Ra1 values of the respective group. On the contrary, at Ra2, all the specimens (Figure 6A1–E1) demonstrated variations in the form of deep scores and uneven surfaces, demonstrating increased roughness due to brushing.

**Figure 7:**
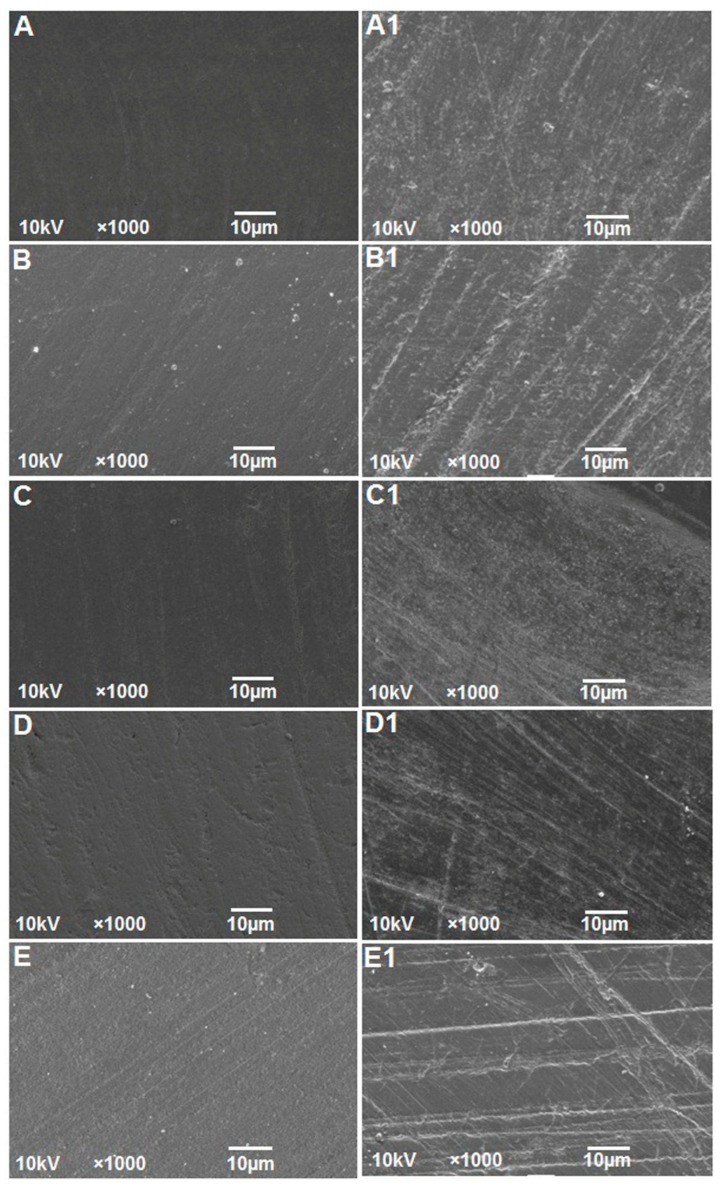
Representative SEM micrographs of the study groups obtained before (**A**–**E**) and after coffee staining and mechanical brushing (**A1**–**E1**). (**A**,**A1**)-Conventional VFRs; (**B**,**B1**)-3D-printed—0°; (**C**,**C1**)-3D-printed—15°; (**D**,**D1**)-3D-printed—30°; (**E**,**E1**)-3D-printed—45°.

SEM photomicrographs of the representative conventional and 3D-printed specimen groups are presented in Figure 7. The SEM micrographs showed significant changes from Ra1 (Figure 7A–E) to Ra2 (Figure 7A1–E1). The surface topographic changes were consistent with that of the profilometric images of the groups. Surface changes at Ra2 in a series of grooves were evident on all the sample surfaces. The 3D-printed (0°, 30°, and 45°) specimens showed more prominent topographic changes compared to the other two groups.

## 4. Discussion

This in vitro study aimed to evaluate the influence of printing angulation, coffee staining, simulated brushing-induced color changes, and surface roughness of 3D-printed orthodontic retainers to compare the outcome with the conventional thermoplastic orthodontic retainer. It was hypothesized that there would be no significant difference in ΔE and Ra between the 3D-printed retainers and conventional VFRs. The study’s results demonstrated a significant difference (*p* < 0.01) in the ΔE between conventional VFRs and the 3D-printed-45° group (*p* < 0.01) only, and the mean Ra of the conventional VFRs was significant (*p* < 0.01) with the 3D-printed groups except for 3D-printed-15° group (*p* > 0.01). This outcome suggests partial rejection of the first null hypothesis.

Long-term retainer use is the only method that prevents orthodontic relapse and produces a stable outcome following orthodontic treatment [2,9]. Due to the aesthetic aspects and reasonable treatment times, clear retainers have become popular as orthodontic procedures have advanced. These clear retainers must be maintained to prevent loss of material integrity because interactions between clear retainer material and the oral environment can result in plaque and calculus formation and bacteria buildup and retention on the retainer surface [50]. In the current study, copolyester thermoplastic sheets were used to fabricate the conventional VFRs and compare them with 3D-printed retainers. Copolyester is a result of the modifications of polyester, such as polyethylene terephthalate, with isophthalic acid or other diols. They are shown to wear less and are more transparent than polypropylene polymers [51].

Color stability is an important property for a dental appliance, especially for clear or esthetic appliances. The color is affected by intra-oral aging, ultraviolet radiation, mouthwash, and various dietary beverages [50]. In this study, the retainers were immersed in coffee beverages, partly due to its chromogenic nature of inducing discoloration and because it is a commonly consumed beverage worldwide [17]. The perceptibility threshold (PT) and the acceptability threshold (AT) are crucial for evaluating ΔE. The ΔE value of ≤1 is visually undetectable in a typically controlled environment. Per the ISO/TR-28642:2016 norms, the PT (ΔE ≤ 1.2) and AT (ΔE = 1.2–2.7) were used to differentiate the ΔE values in this study. Clinically unacceptable ΔE values were those that exceeded the AT limit (ΔE ≥ 2.7) [17].

The comparison of the mean ∆E values showed that ∆E of conventional VFR was between the PT and AT (∆E = 2.18), and the ∆E of 3D-printed groups (irrespective of the printing angulation) was above AT (∆E > 2.7). The obtained ∆E values were converted to NBS units in relating the ∆E values to clinical conditions. Among the groups, conventional VFR and 3D-printed-15° demonstrated appreciable color changes (1.5–3.0 NBS units), while the remaining 3D-printed groups showed noticeable color changes (3.0–6.0 NBS units). The difference in ∆E between the conventional and 3D-printed groups may be due to the 3D-printed resins’ surface degradation, which may have affected ∆E. Surface degradation and filler content are inversely correlated, and most 3D-printed resins have less inorganic fillers [17]. On the contrary, excellent transparency, appropriate flow properties, durability, and high chemical change resistance of the copolyester retainer materials could have contributed to higher color stability than 3D-printed groups [52].

The outcome of the study also demonstrated that an increase in surface roughness (Ra2) of the specimens presented with increased color changes. This correlation could be possibly explained by the surface properties of the materials, such as roughness, which accelerate pigment deposition and staining on the specimen surface, thus implicating the differences in the color changes [53]. In order to avoid the accumulation of plaque and bacteria, dental materials should have mean Ra values below 0.2 µm [54]. Plaque accumulates more quickly when surface roughness increases over time. According to studies, if the roughness value surpasses 0.5 µm, an intraoral hard surface can be uncomfortable and felt by the human tongue [55]. The profilometer is the most popular method among dental researchers for measuring surface roughness since it provides a quantitative assessment of surface topography. The common roughness parameter in general quality control is the average arithmetic height (Ra), which is easy to define and compute and gives a reasonable idea of height variations. However, still, comprehensive specimen surface details were provided in this study by combining quantitative measures with qualitative SEM examination [35].

While the current tested materials underwent the same finishing and polishing procedure, there was no discernible difference between them at Ra1. However, roughness (Ra2) significantly increased above the clinical perceptibility threshold (Ra = 0.5 µm) following immersion in coffee beverages and simulating brushing. Toothbrush abrasion is used to evaluate the behavior of dental materials by analyzing the surface changes induced by brushing in an experimental setting [56]. The most popular, easy, and cost-effective mechanical approach to plaque control by an individual is tooth brushing with dentifrice. Contrarily, using dentifrice to brush has been associated with roughening the surfaces of dental hard tissues and restorative and prosthetic dental materials [56]. According to the Relative Dentin Abrasivity (RDA), the Colgate Regular dentifrice used in this research has a mild abrasive (RDA = 68) action. Consequently, it is unrealistic to imply that the low abrasive toothpaste impacted the obtained Ra2 values [35].

The roughness changes could be related to hydrolytic degradation when polymer materials absorb water or other liquid from the atmosphere. Water or beverages and the polymer matrix react chemically during hydrolysis, changing the structure and characteristics of the polymer matrix in many ways. The polymers are irreversibly damaged, and all these modifications impact material roughness [48]. Furthermore, other factors such as material structure, composition and properties, liquid polarity, exposure time of liquids, and pH could all influence the surface roughness of the materials. The increase in surface roughness from Ra1 to Ra2, as confirmed by the profilometer, is well corroborated with the SEM micrographs. The outcome of the study also demonstrated that an increase in surface roughness (Ra2) of the specimens correlated with increased color changes.

The second null hypothesis of this study was that the four different printing angulations (0°, 15°, 30°, and 45°) would present no significant differences in the ΔE and Ra when printing orthodontic retainers. The study’s outcome demonstrated no significant difference in ΔE among the four printing angulations. Nonetheless, the Ra2 of the 3D-printed-15° group was significant with other 3D-printed groups (*p* < 0.01), which suggests partial rejection of the second null hypothesis.

For obtaining precision, smoothness, and accuracy when printing dental models, a print angulation of <30 is suggested by the manufacturer [14,37]. Nonetheless, the configuration must be positioned as nearly vertically as feasible in order to fabricate the maximum number of models efficiently during one print cycle [14]. In general, the shape and printing direction affect the number of models that can be planted on a platform; vertical printing can create more models than horizontal printing [16]. Previous studies have shown mixed outcomes with regard to the accuracy of print angulations.

Williams et al. [14] investigated the precision and accuracy of 3D-printed retainers at different angulations (15°, 30°, 45°, 60°, and 90°) and the impact of angulation on printing time and resin consumption. When compared to the digital reference file, it was found that 3D-printed retainers were accurate to within 0.25 mm at all print angulations at the cusp tips and incisal edges. Smooth facial surfaces extended beyond clinically acceptable levels. The most time and money-efficient printing angulations were 15 and 45 degrees, respectively. McCarty et al. [40] evaluated the impact of print orientation (horizontal, vertical, and 45°) on the dimensional accuracy of 3D-printed clear aligners. Under the testing conditions, the authors concluded that print orientation had no significant effect on the overall accuracy of the 3D-printed aligner design. Boyer et al. [41] investigated the effect of print orientation (0°, 45°, 90°, 135°, 180°, 225°, 270°, and 315°) on the dimensional accuracy of 3D-printed orthodontic aligners. Printing at 90° angulation provided the most accurate prints compared to the other seven orientations tested, albeit not all differences were statistically significant.

In this study, 15° print angulation was efficient in providing specimens with high color stability and low roughness compared to other angulations. A 45° print angulation provided low color stability and high roughness values. Shim et al. [36] evaluated the roughness of 3D-printed PMMA specimens in three printing orientations (0°, 45°, and 90°). The authors found significantly low roughness values for specimens printed at 0° and 90° compared to specimens printed at 45°.

To the authors’ knowledge, this is the first study to evaluate the color and roughness of 3D-printed retainer materials in four different printing angulations. Although the study outcome fills an important aspect in the literature related to the color changes and roughness of 3D-printed retainer materials, this study has a few limitations. Foremost, the in vitro protocol of the study is the limitation, despite the intra-oral simulation being as close as possible. The surface treatment in this study followed a one-after-the-other approach (e.g., immersion followed by brushing), whereas this process is simultaneous in the oral cavity. The influence of intra-oral conditions could present a more deviated outcome considering the actions of saliva, personalized oral hygiene habits, and diet. The exact composition of the 3D-printed resins is yet to be available due to the manufacturers’ trade secrets, which hinders drawing conclusions about the observed differences. The specimens were prepared flat and did not follow the retainer shape, another limitation of this study. Data regarding the color and roughness of 3D-printed orthodontic retainer materials are scarce, making the comparison of the present result with previous data challenging. 

A previous study has shown that the long-term flexibility of the retainer materials is influenced by toothbrushing [51]. Hence it is imperative to study the flexural strength and modulus of the 3D-printed retainer materials at different angulations after toothbrushing. The 3D-printed retainer materials’ anti-microbial activity related to printing directions should be tested. Furthermore, the effect of different beverages on the 3D-printed retainer materials could provide a broader understanding of the color and roughness results. It is also worth studying the action of different chemical cleansers or destaining agents on the color and roughness of the 3D-printed retainer materials.

## 5. Conclusions

Considering the study’s limitations, the following conclusions are made:

The overall color stability of 3D-printed orthodontic retainers significantly differed from that of conventional VFRs. The conventional and 3D-printed-15°groups demonstrated appreciable color changes, while the other 3D-printed groups showed noticeable color changes per the NBS units.

The surface roughness of 3D-printed orthodontic retainers was not comparable to that of conventional VFRs, except for the 3D-printed-15° group after coffee immersion and simulated brushing. The conventional and 3D-printed groups exceeded the clinical perceptible roughness threshold (Ra > 0.5 µm) after coffee immersion and simulated brushing.

Among the different angulations used to print the 3D-printed retainers, the 15° angle was the most efficient in terms of color stability and surface roughness and was comparable to conventional VFRs.

## Figures and Tables

**Table 1:** National Bureau of Standard (NBS) units inference.

NBS Units	Inference of Color Change
0.0–0.5	Trace: extremely slight change
0.5–1.5	Slight: slight change
1.5–3.0	Appreciable: marked change
3.0–6.0	Noticeable: perceivable
6.0–12.0	Much: extremely marked change
>12.0	Very much: change to another color

**Table 2:** Mean pair-wise comparison of ΔE among the study groups.

Group (I)	Group (J)	Mean Difference (I-J)	*p*-Value	95% CI for Mean Diff	
				Lower Bound	Upper Bound
Conventional VFRs	3D-printed—0°	−1.446	0.381	−3.44	0.55
	3D-printed—15°	−0.659	1.000	−2.66	1.34
	3D-printed—30°	−1.114	1.000	−3.11	0.88
	3D-printed—45°	−2.498	0.006 *	−4.50	−0.50
3D-printed—0°	3D-printed—15°	0.787	1.000	−1.21	2.78
	3D-printed—30°	0.332	1.000	−1.67	2.33
	3D-printed—45°	−1.052	1.000	−3.05	0.95
3D-printed—15°	3D-printed—30°	−0.455	1.000	−2.45	1.54
	3D-printed—45°	−1.839	0.093	−3.84	0.16
3D-printed—30°	3D-printed—45°	−1.384	0.467	−3.38	0.61

* Statistically significant (*p* < 0.01).

**Table 3:** Overall mean comparison of ΔE between conventional VFRs and 3D-printed groups.

Group	Mean	SD	SE of Mean	Mean Difference	t	*p*-Value
Conventional VFRs	2.18	0.87	0.28	−1.429	−4.003	0.001 *
3D Printed	3.61	0.72	0.23			

* Statistically significant (*p* < 0.05).

**Table 4:** Pairwise comparison of Ra2 among the study groups.

Group (I)	Group (J)	Mean Difference (I-J)	*p*-Value	95% CI for Mean Diff	
				Lower Bound	Upper Bound
Conventional VFRs	3D-printed—0°	−0.188	0.001 *	−0.32	−0.05
	3D-printed—15°	−0.020	1.000	−0.15	0.11
	3D-printed—30°	−0.219	<0.001 *	−0.35	−0.09
	3D-printed—45°	−0.266	<0.001 *	−0.40	−0.13
3D-printed—0°	3D-printed—15°	0.168	0.006 *	0.03	0.30
	3D-printed—30°	−0.031	1.000	−0.17	0.10
	3D-printed—45°	−0.078	0.935	−0.21	0.06
3D-printed—15°	3D-printed—30°	−0.200	0.001 *	−0.33	−0.07
	3D-printed—45°	−0.246	<0.001 *	−0.38	−0.11
3D-printed—30°	3D-printed—45°	−0.047	1.000	−0.18	0.09

* Statistically significant values (*p* < 0.05).

**Table 5:** Mean comparison of Ra from Ra1 to Ra2.

Group	Treatment	Mean	SD	SE of Mean	Mean Difference	t	*p*-Value
Conventional VFRs	Ra1	0.26	0.07	0.02	−0.482	−12.956	<0.001 *
	Ra2	0.74	0.12	0.04			
3D-printed—0°	Ra1	0.26	0.04	0.01	−0.667	−25.745	<0.001 *
	Ra2	0.93	0.07	0.02			
3D-printed—15°	Ra1	0.23	0.08	0.03	−0.534	−17.126	<0.001 *
	Ra2	0.76	0.06	0.02			
3D-printed—30°	Ra1	0.23	0.10	0.03	−0.728	−20.769	<0.001 *
	Ra2	0.96	0.10	0.03			
3D-printed—45°	Ra1	0.29	0.05	0.02	−0.721	−14.541	<0.001 *
	Ra2	1.01	0.13	0.04			

* Statistically significant values (*p* < 0.05).

**Table 6:** Overall mean comparison of Ra1 and Ra2 between conventional VFRs and 3D groups.

Treatment	Group	Mean	SD	SE of Mean	Mean Difference	t	*p*-Value
Ra1	Conventional VFRs	0.26	0.07	0.02	0.006	0.227	0.824
	3D-Printed	0.25	0.03	0.01			
Ra2	Conventional VFRs	0.74	0.12	0.04	−0.173	−4.318	0.001 *
	3D-Printed	0.92	0.05	0.02			

* Statistically significant values (*p* < 0.05).

## Data Availability

Data sharing is not applicable to this article.
